# Microplastics in food production systems: Sources, exposure pathways, and gastrointestinal health effects

**DOI:** 10.1016/j.crfs.2026.101557

**Published:** 2026-09-08

**Authors:** Zahra Beyzaei, Ralf Weiskirchen

**Affiliations:** a Transplant Research Center, Shiraz University of Medical Sciences, Shiraz, Iran; b Institute of Molecular Pathobiochemistry, Experimental Gene Therapy and Clinical Chemistry (IFMPEGKC) RWTH University Hospital Aachen, Aachen, D-52074, Germany

**Keywords:** Microplastics, Nanoplastics, Human gut, Gastrointestinal toxicity, Metabolic dysregulation, Human health risk assessment

## Abstract

Microplastics (MPs) and nanoplastics (NPs) have become ubiquitous contaminants in food production systems, raising increasing concerns regarding their potential effects on gastrointestinal and systemic health. This review summarizes current evidence on the occurrence, sources, and biological effects of MPs/NPs within food production chains and their implications for the human gut. Experimental *in vitro* studies demonstrate that MPs/NPs can induce oxidative stress, inflammation, genotoxicity, metabolic dysregulation, and epithelial barrier dysfunction, while *in vivo* animal studies show disruption of gut homeostasis, microbiota alterations, systemic translocation, and hepatic, metabolic, and immune disturbances. Co-exposure to environmental pollutants and food-associated chemicals may further enhance toxicity through synergistic mechanisms. Although human evidence remains limited, MPs have been detected in human tissues, including the liver, indicating the possibility of systemic exposure. Emerging evidence also suggests complex interactions between MPs/NPs and the gut microbiome, although the clinical significance of these findings remains uncertain. Therefore, current knowledge is derived predominantly from experimental models, and substantial uncertainties remain regarding human exposure levels, dose-response relationships, and long-term health consequences. Further well-designed human studies and standardized exposure assessment methods are essential to clarify the health risks associated with dietary MPs/NPs.

## Highlights

• Microplastics/nanoplastics enter food via farming, processing, packaging.
• Food microplastics/nanoplastics create safety and dietary risk concerns.
• Microplastics/nanoplastics may harm gut barriers and raise oxidative stress.
• Dysbiosis and liver translocation may cause systemic harm.
• Limited human data reveal major research gaps.

## Graphical abstract

**Figure:**
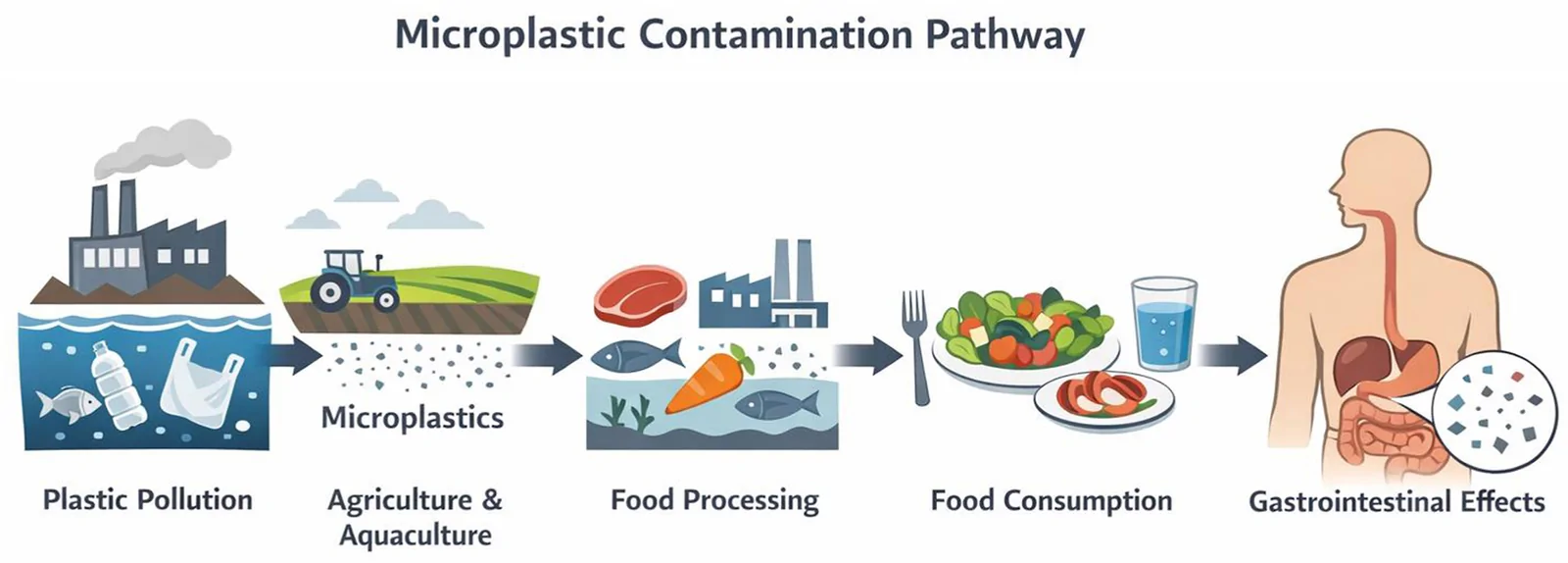

## 1 Introduction

Microplastics (MPs), synthetic polymer fragments smaller than 5 mm in diameter, have emerged as critical contaminants within global food systems, raising substantial concerns for food safety and public health (Bastyans et al., 2022). These degraded plastic particles now pervade drinking water, agricultural soils, and both aquatic and terrestrial organisms, establishing food consumption as the predominant route of human exposure (Xiao et al., 2026).

To systematically characterize their environmental distribution and toxicological profiles, MPs are classified according to both size and polymer composition. Size-based categorization distinguishes among macroplastics, MPs, and nanoplastics (NPs), while chemical classification identifies six principal polymer types: polyethylene terephthalate (PET), polymethyl methacrylate (PMMA), polyvinyl chloride (PVC), polypropylene (PP), polyoxymethylene (POM), and polystyrene (PS) (Adamu et al., 2024). Each polymer exhibits distinct physicochemical properties that influence environmental persistence, pollutant adsorption capacity, and biological interactions (Beyzaei et al., 2025).

MPs are introduced into the human body through multiple pathways, including contaminated agricultural products, seafood, dairy products, and packaging materials, raising serious concerns regarding their potential effects on human health. Aquatic ecosystems represent a particularly critical nexus in this contamination pathway. For millennia, wild fisheries have provided essential protein and micronutrients to human populations worldwide (Hassoun et al., 2023). The rapid expansion of aquaculture over recent decades, now among the fastest-growing food production sectors, was intended to alleviate pressure on declining wild fish stocks and address overfishing (Uddin et al., 2026; Naser et al., 2026). However, both wild-capture and farmed aquatic food systems face mounting sustainability challenges from anthropogenic pollution, and MP contamination is increasingly recognized as a potential and growing concern, although its long-term implications for food system sustainability remain to be fully quantified (Dong and Wang, 2026). Current evidence indicates that MPs can undergo trophic transfer across food webs, resulting in measurable microplastic burdens in edible organisms and, ultimately, human consumers. However, whether MPs undergo true biomagnification remains uncertain and requires further quantitative investigation (Karak et al., 2025).

The toxicological consequences of MP exposure have been investigated predominantly in *in vitro* and animal models and are suggested to involve multiple physiological systems. Among the earliest and most direct targets of MP exposure is the gastrointestinal (GI) system. Experimental studies have demonstrated that MPs may induce gastrointestinal inflammation, hepatocyte injury, disruption of gut microbial communities, and perturbations of respiratory, reproductive, nervous, and circulatory functions (Beyzaei et al., 2025; Singh et al., 2026). However, direct evidence linking dietary MP exposure to these adverse health outcomes in humans remains limited, and causal relationships have not yet been firmly established. In addition, current understanding remains limited regarding the mechanisms by which MP-induced GI damage propagates through interorgan communication networks to disrupt systemic physiological homeostasis.

Although numerous recent reviews have summarized the environmental occurrence, analytical detection, or general toxicological effects of MPs, most have considered individual organs or biological processes in isolation. Few have comprehensively integrated dietary exposure pathways with gastrointestinal toxicity, gut microbiota dysbiosis, gut–liver axis dysfunction, and their broader implications for systemic health and food safety. Accordingly, the novelty of this review lies in providing a mechanism-based synthesis that links dietary MP exposure to GI injury and subsequent cross-organ physiological responses through the gut–liver axis. By critically integrating evidence from experimental and emerging human studies, identifying mechanistic knowledge gaps, and discussing implications for food safety and future risk assessment, this review offers a broader conceptual framework that extends beyond descriptive summaries of microplastic toxicity.

Therefore, this review aims to guide future mechanistic research, improve understanding of the health consequences of dietary MP exposure, and support the development of evidence-based strategies to protect GI health in an increasingly plastic-contaminated food environment.

## 2 Microplastics in food production systems

**Fig. 1:**
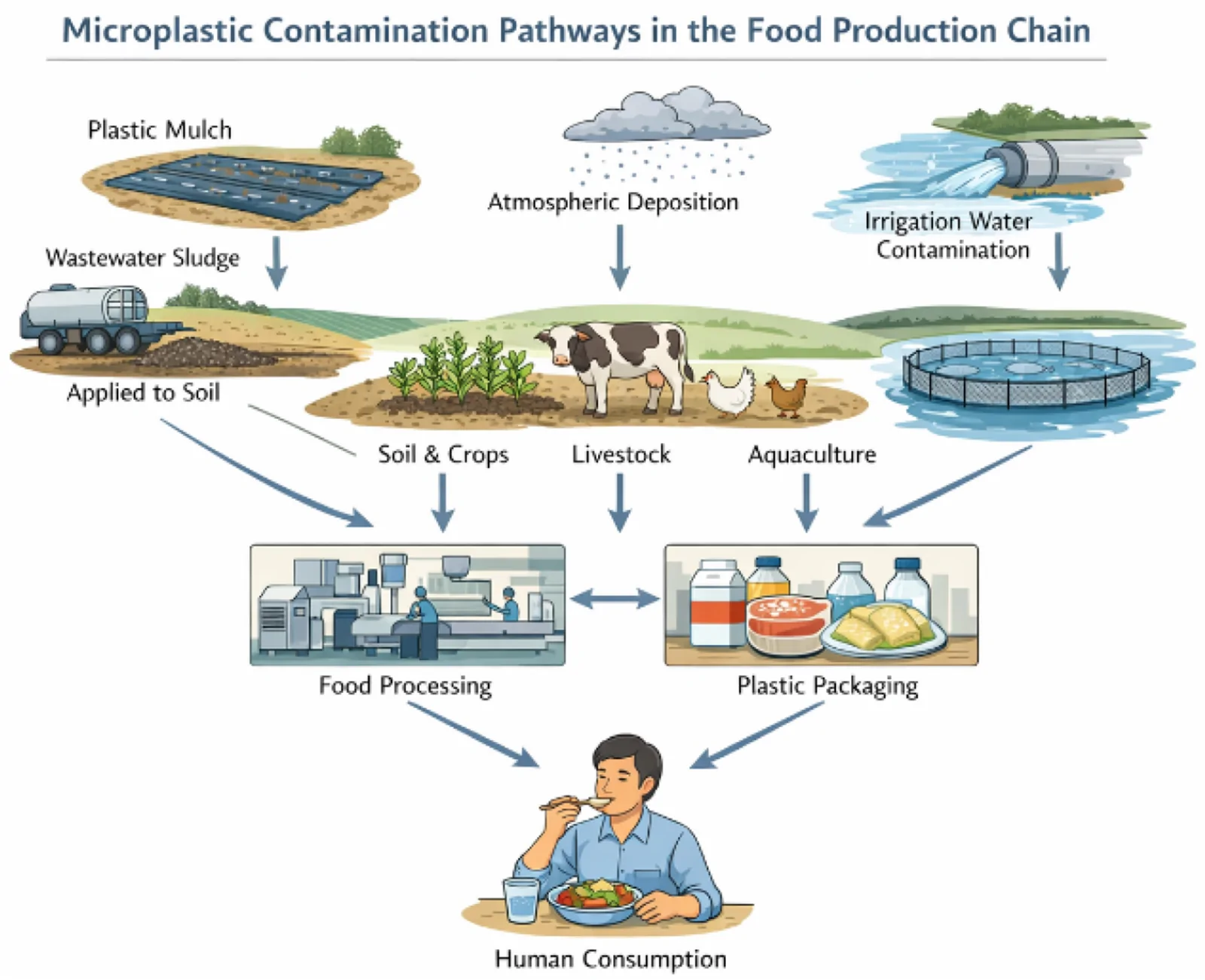
**Pathways introducing microplastics into the food production chain.** Microplastics originate from multiple environmental sources, including plastic mulch degradation, wastewater sludge applied to agricultural soils, atmospheric deposition, and contaminated irrigation water. These particles enter primary production systems such as crops, livestock, and aquaculture, and may be further introduced during food processing and through plastic packaging materials. Collectively, these pathways contribute to the accumulation of microplastics in food products and their subsequent ingestion by humans.

MPs have emerged as pervasive contaminants in terrestrial, aquatic, and atmospheric environments, raising increasing concerns for ecosystem stability, food safety, and human health (Yaghoubi Khanghahi et al., 2026). MPs primarily originate from the fragmentation and degradation of larger plastic materials, as well as from industrial, agricultural, and domestic activities. Due to their persistence and mobility, MPs are continuously introduced into environmental systems through wastewater discharge, atmospheric deposition, urban runoff, and agricultural practices, resulting in their accumulation in soils, freshwater bodies, marine ecosystems, and the atmosphere (Fig. 1).

In food production systems, agricultural activities represent a major pathway for MP contamination. The extensive use of plastic mulching films, sewage sludge, organic fertilizers, and wastewater irrigation contributes significantly to MP accumulation in agricultural soils (Xiao et al., 2026; Yaghoubi Khanghahi et al., 2026). In many regions, MP concentrations in soil have been reported to range from several hundred to more than 13,000 particles kg^−1^ dry soil, depending on land use patterns and local agricultural or industrial practices (Zhu et al., 2026; Zhang et al., 2026a). Once present in soil, MPs may interact with soil microorganisms, alter physicochemical properties, and interfere with nutrient cycling and plant growth (Vleeming et al., 2026). Recent studies further indicate that MPs can adhere to plant surfaces or penetrate root tissues, facilitating their transfer into edible crops (Zhu et al., 2026; Vleeming et al., 2026; Rillig et al., 2019; Leslie et al., 2017). Root vegetables appear particularly vulnerable because roots are in direct contact with contaminated soils, raising concerns regarding human exposure through dietary intake (Vleeming et al., 2026). Recent studies have also demonstrated the occurrence of MPs in plant-derived food products. For example, one study reported the presence of MPs in extra-virgin olive oil, a key component of the Mediterranean diet, raising concerns regarding contamination of nutritionally valuable foods and potential implications for food quality and safety (Vita et al., 2026).

**Fig. 2:**
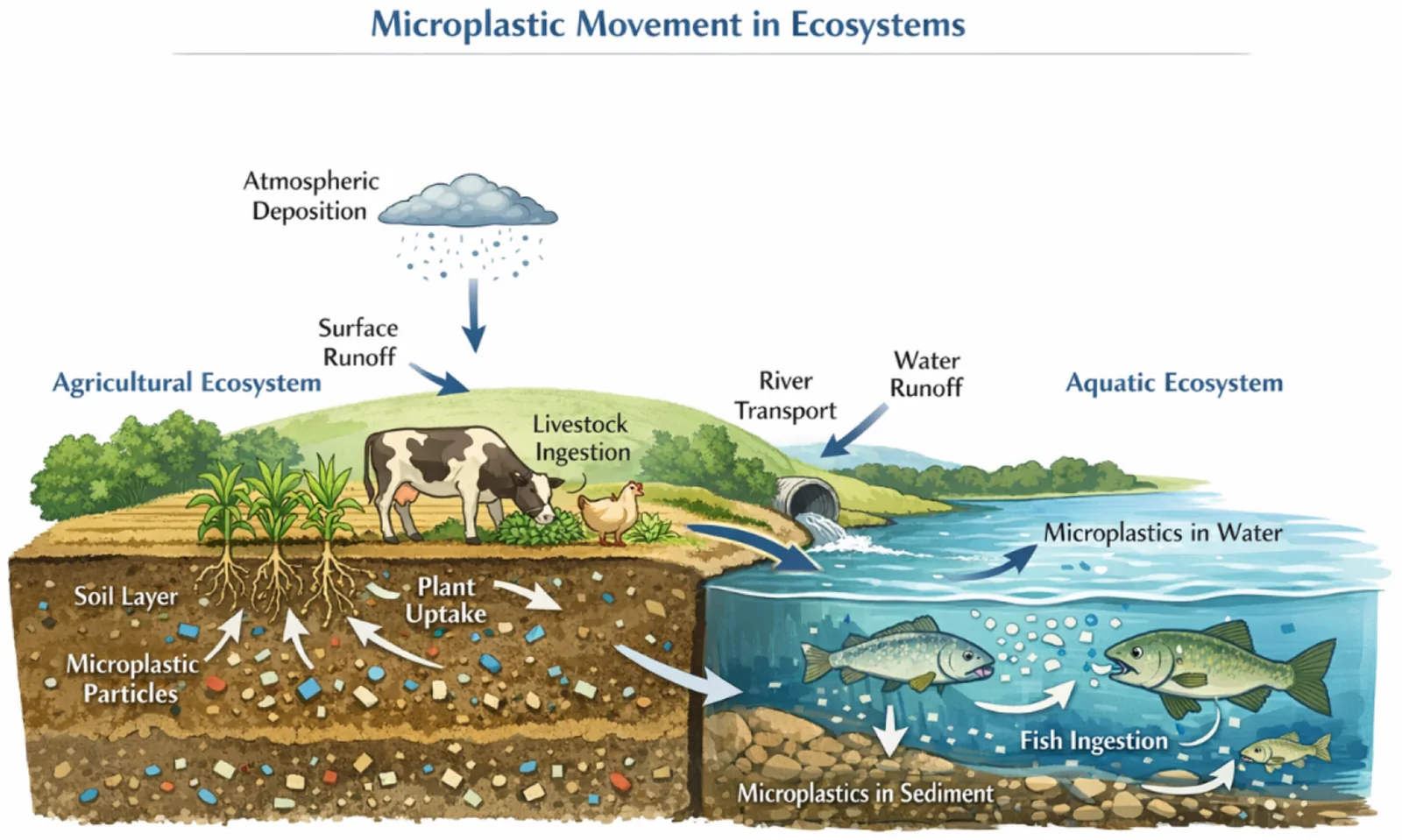
**Environmental transport of microplastics through agricultural and aquatic systems.** Microplastics deposited from the atmosphere or introduced into soils accumulate in the soil layer, where they may be taken up by plants or ingested by livestock. Surface runoff and river transport subsequently transfer these particles into aquatic environments, where they persist in the water column and sediments. Aquatic organisms, including fish, ingest microplastics, facilitating their movement through food webs and contributing to their eventual entry into the human food chain.

Animal production systems constitute another major pathway for MPs entering the food chain (Fig. 2). Livestock and aquatic organisms are exposed to MPs through contaminated feed, drinking water, and surrounding environmental matrices (Uddin et al., 2026; Rahman et al., 2026). Following ingestion, MPs and their associated chemical contaminants may accumulate in tissues and organs, thereby facilitating their transfer into animal-derived food products, including meat, milk, eggs, and seafood (Naser et al., 2026; Mitu et al., 2026; Dong et al., 2026; Bristi and Islam, 2026; Visentin et al., 2025; Thamsenanupap et al., 2026). Thus, these findings underscore the capacity of MPs to undergo trophic transfer across food webs, contributing to continuous and chronic human exposure through dietary intake. Although numerous studies have demonstrated trophic transfer of MPs along food chains, evidence supporting true biomagnification remains limited and inconclusive. Therefore, current evidence is more appropriately interpreted as trophic transfer resulting in measurable MP burdens in edible organisms and, ultimately, human consumers rather than unequivocal biomagnification.

Aquaculture has become one of the fastest-growing sectors of food production, playing a critical role in global food security, economic development, and the supply of animal protein. However, intensive aquaculture practices may also contribute to environmental degradation and food safety concerns. Discharges from aquaculture systems often contain elevated levels of nutrients, organic matter, chemical residues, and MPs, which can adversely affect surrounding aquatic ecosystems. These contaminants may accumulate within cultured organisms and subsequently enter the human food chain, raising increasing concerns regarding ecological sustainability and public health (Dong and Wang, 2026; Xu et al., 2025). Fish are widely recognized as important bioindicators of aquatic environmental contamination because they can accumulate pollutants from surrounding water, sediments, and dietary sources. In addition to their ecological significance, fish constitute a major component of the human diet worldwide owing to their high nutritional value, including high-quality proteins, low levels of saturated fats, and abundant omega-3 polyunsaturated fatty acids. The accumulation and distribution of contaminants in fish and shellfish are influenced by several factors, including species, age, habitat, geographic origin, seasonal variation, feeding behavior, and tissue type. Consequently, substantial variability in contaminant burdens has been reported both within and among wild and farmed aquatic species (Uddin et al., 2026; Rose, 2026).

**Fig. 3:**
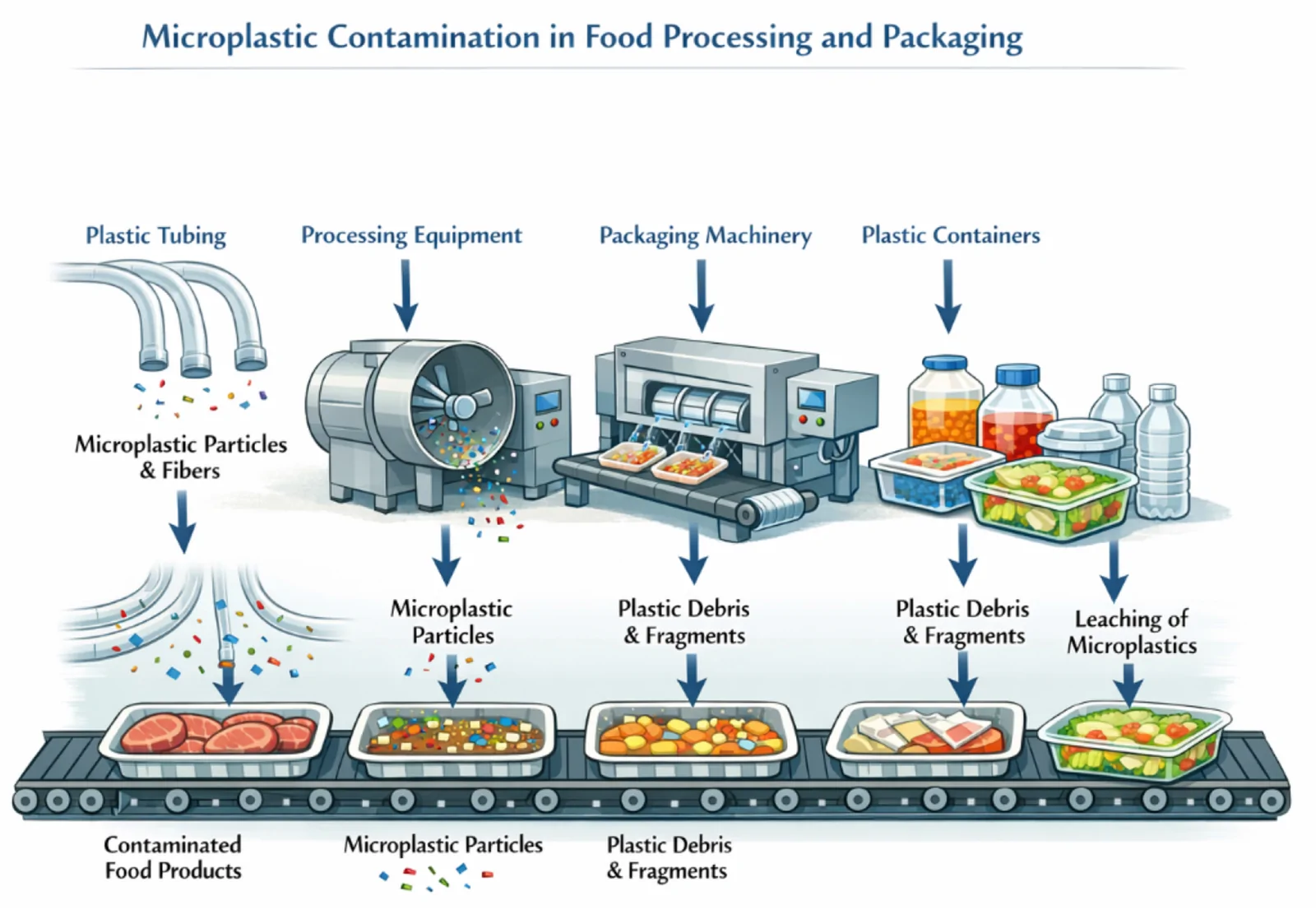
**Potential introduction of microplastics during food processing and packaging.** Microplastics can be introduced into food products at multiple stages of industrial processing. Potential sources include plastic tubing, processing equipment, and packaging machinery, which may release microplastic particles, fibers, or plastic fragments through mechanical abrasion and material degradation. In addition, plastic containers and packaging materials can contribute to contamination through the release or leaching of microplastics, leading to their accumulation in processed food products.

In addition to primary production, food processing and packaging operations constitute secondary sources of MP contamination (Fig. 3). Abrasion and mechanical wear of plastic processing equipment, conveyor belts, cutting boards, synthetic filtration systems, and packaging materials can release MPs into food products during industrial handling, transportation, and storage (Zhang et al., 2026b). Thermal processing and mechanical stress may further accelerate particle release from plastic containers, bottles, wraps, and other food-contact materials (Qu et al., 2026). However, reported concentrations of MPs released from food-contact materials vary considerably among studies because of differences in packaging materials, experimental conditions, food matrices, and analytical methodologies (Dong et al., 2026; Duda and Petka, 2025). Therefore, the relative contribution of food packaging and processing equipment to total dietary MP exposure has not yet been fully quantified and remains an active area of investigation. Consequently, MPs have been increasingly detected in a wide range of food products, including vegetables, fruits, seafood, processed foods, dairy products, and drinking water (Muñoz-Lapeira and Balcázar, 2026).

For example, tea bags have been identified as a significant source of MPs/NPs, often releasing substantially higher concentrations than many other beverages and food products (Naser et al., 2026). This emerging issue has raised concerns regarding the potential implications for both human health and environmental sustainability, highlighting the need for further investigation (Naser et al., 2026). Different beverage packaging materials represent important potential sources of MP/NP contamination. Cold beverages are commonly packaged in plastic containers, primarily PET bottles, or in aseptic cartons containing inner plastic linings, typically composed of low-density polyethylene (LDPE). Similarly, tea bags are manufactured from a variety of materials, including biodegradable plant fibers, polylactic acid (PLA), petroleum-based woven or non-woven plastics such as PP, nylon-6/nylon-6,6, and PET, as well as cellulose–plastic composites. During preparation and storage, these materials can release MPs/NPs into beverages, thereby contributing to human dietary exposure (Fard et al., 2025; Lin et al., 2022).

The relative contribution of different food production stages to human dietary MP exposure remains difficult to quantify because contamination originates from multiple interconnected sources and varies according to food type, production practices, and analytical methodology. Current evidence suggests that primary production systems, including agriculture and aquaculture, represent major sources of environmental contamination, whereas food processing, packaging materials, and food-contact equipment may contribute additional secondary contamination during manufacturing, storage, and preparation (Prata et al., 2020). However, the magnitude of each source differs among food commodities, and no consensus has yet been reached regarding their relative contribution to overall dietary MP intake. Standardized exposure assessment studies are therefore needed to identify the dominant contamination pathways and support effective risk management strategies.

Therefore, the widespread occurrence of MPs throughout food production systems demonstrates their capacity to migrate from environmental reservoirs into the human food chain. Given their persistence, bioavailability, and ability to adsorb toxic chemicals and pathogenic microorganisms, MPs are increasingly recognized as emerging contaminants of global concern, necessitating further investigation into their environmental fate, food safety implications, and long-term health risks.

## 3 Dietary exposure and risk assessment

Dietary intake represents the dominant route of human exposure to MPs and NPs, with contamination levels varying substantially across food matrices, geographic regions, and food processing methods. Among beverages, tap and bottled water, beer, and milk show measurable contamination, while processed foods such as refined sugar have recently been shown to harbor a substantial fraction of particles below 20 μm, a size range not yet fully addressed by current regulatory frameworks (Hayder et al., 2026; Fadda et al., 2026). Plastic-wrapped confectionery has also emerged as an underrecognized source, with estimated daily intakes of 23-25 particles/kg body weight/day in children aged 1-5 years, raising particular concern given the vulnerability of this age group (Adjama and Dave, 2026). At a broader dietary level, comparative analyses across 13 food and beverage categories indicate that although seafood has historically dominated the research literature, fruit, vegetables, and grains contribute the highest estimated daily intake once actual consumption patterns are accounted for, with total daily intake estimates spanning several orders of magnitude (7.7× 10^−3^ to 3.8 × 10^8^ particles/kg bw/day; median 721 particles/kg bw/day) (Hayder et al., 2026). Dietary pattern itself further modulates exposure: lacto-ovo-vegetarian diets have been associated with nearly double the MP intake of Mediterranean or Western dietary patterns, owing to higher consumption of fruit, vegetables, legumes, and nuts, even though the Mediterranean diet is considered the healthiest overall when nutritional benefits are weighed against microplastic burden (Duda and Petka, 2025). Considerable uncertainty persists in these exposure estimates, stemming from inconsistent analytical techniques, such as Fourier-transform infrared (FTIR) spectroscopy and μ-Raman spectroscopy, a lack of harmonized detection thresholds for small particles, and limited data disaggregated by consumer subgroup, including children, pregnant women, and populations with high seafood or bottled water consumption.

Although current evidence demonstrates widespread dietary exposure to MPs, the magnitude of the associated human health risk remains less well characterized than that of established food contaminants such as heavy metals, pesticide residues, or persistent organic pollutants (Krasucka et al., 2022; Lan et al., 2026; Abbasi et al., 2020). It should be emphasized that this comparison is conceptual in nature, reflecting differences in the relative maturity of risk-assessment frameworks rather than a quantitative comparison of risk magnitude. Unlike these contaminants, which have well-defined toxicological profiles, regulatory limits, and standardized monitoring programs, MP risk assessment is complicated by substantial variability in particle size, polymer composition, analytical methodologies, and exposure metrics, together with the absence of health-based guideline values. MPs/NPs, in particular, still lack harmonized exposure metrics, robust dose–response data, and health-based guidance values, unlike many regulated contaminants for which such parameters are already well established. Consequently, while MPs are increasingly recognized as emerging food contaminants of global concern, further harmonization of analytical methods, quantitative exposure assessment, and epidemiological studies is required to place their health risks within the broader context of food safety.

Further studies are needed to generate robust, comparable risk assessments across food matrices and vulnerable populations by addressing current methodological and demographic gaps.

## 4 Gastrointestinal exposure and fate

Considering the diversity of environmental sources and exposure pathways, MPs enter the human body through ingestion, inhalation, and, to a lesser extent, dermal contact (Yuvika et al., 2026). Among these exposure routes, dietary intake through contaminated food, drinking water, and food-contact materials represents the predominant pathway, thereby establishing a direct connection between MP contamination and human health (Oliveri et al., 2020).

**Fig. 4:**
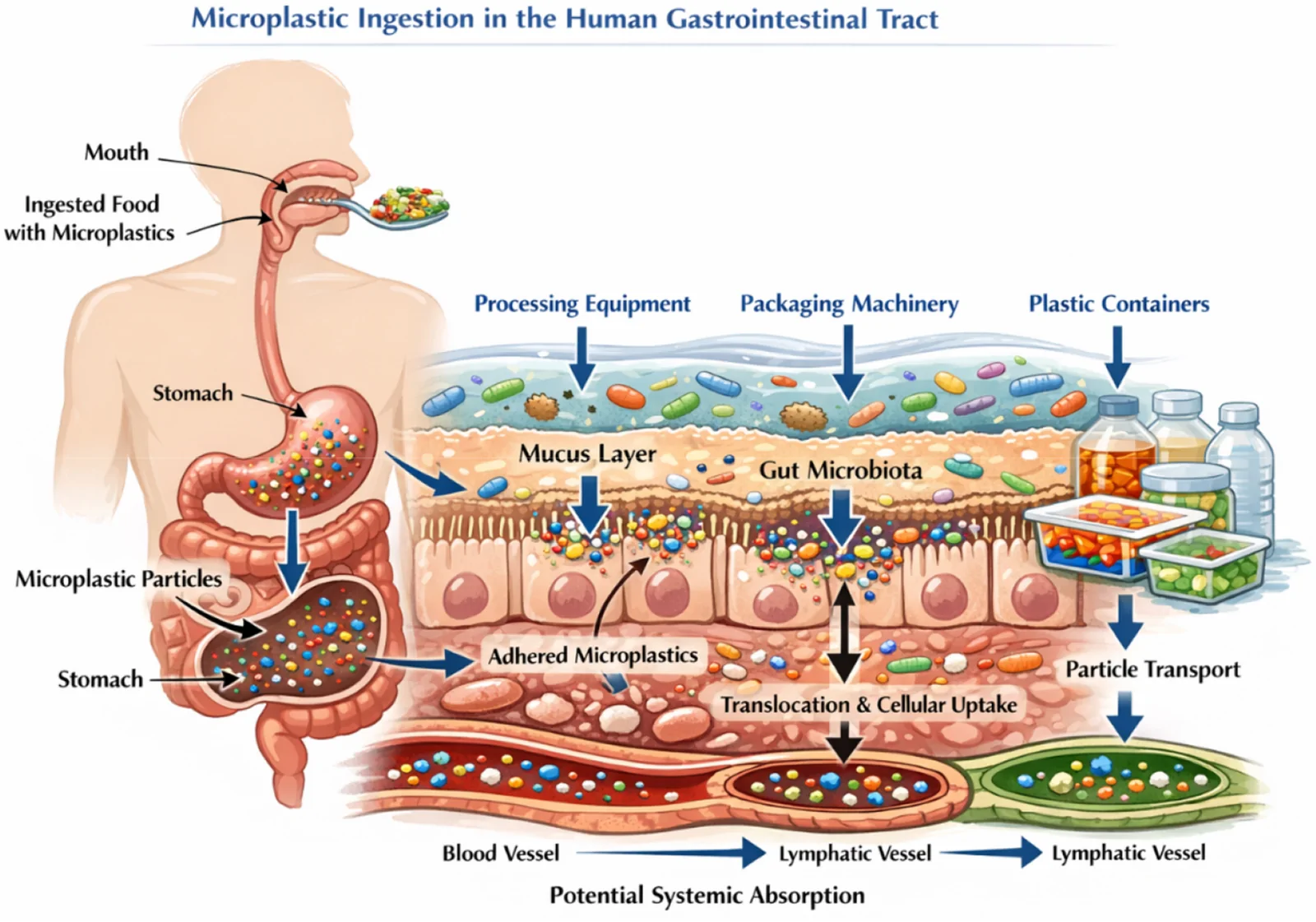
**Microplastic ingestion and interactions within the human gastrointestinal tract.** Microplastics enter the body through contaminated food and beverages and pass through the gastrointestinal tract. Within the intestine, particles interact with the mucus layer and gut microbiota, where some adhere to the epithelial surface. Smaller particles may undergo cellular uptake and translocation across the intestinal barrier, potentially entering blood and lymphatic vessels and contributing to systemic distribution.

Following ingestion, MPs first interact with the GI tract, which serves as the primary interface between external particles and the internal human environment (Fig. 4). The behavior and biological fate of MPs within the digestive system are strongly influenced by their physicochemical characteristics, including particle size, morphology, surface charge, and chemical composition (Bora et al., 2024; Covello et al., 2024). These properties govern their interactions with gastrointestinal fluids, mucus layers, epithelial barriers, and gut microbiota, potentially facilitating cellular uptake and translocation into systemic circulation (Chen et al., 2024).

The human digestive system plays a central role in nutrient digestion and absorption, maintaining physiological homeostasis and overall health. Owing to its continuous exposure to ingested substances, the GI tract is particularly vulnerable to the adverse effects of MPs. Increasing evidence suggests that prolonged exposure to MPs may disrupt intestinal barrier integrity, alter gut microbiota composition, induce oxidative stress and inflammation, and ultimately contribute to both local GI dysfunction and systemic health effects (Yuvika et al., 2026; Wu et al., 2026).

## 5 Gastrointestinal health effects

**Fig. 5:**
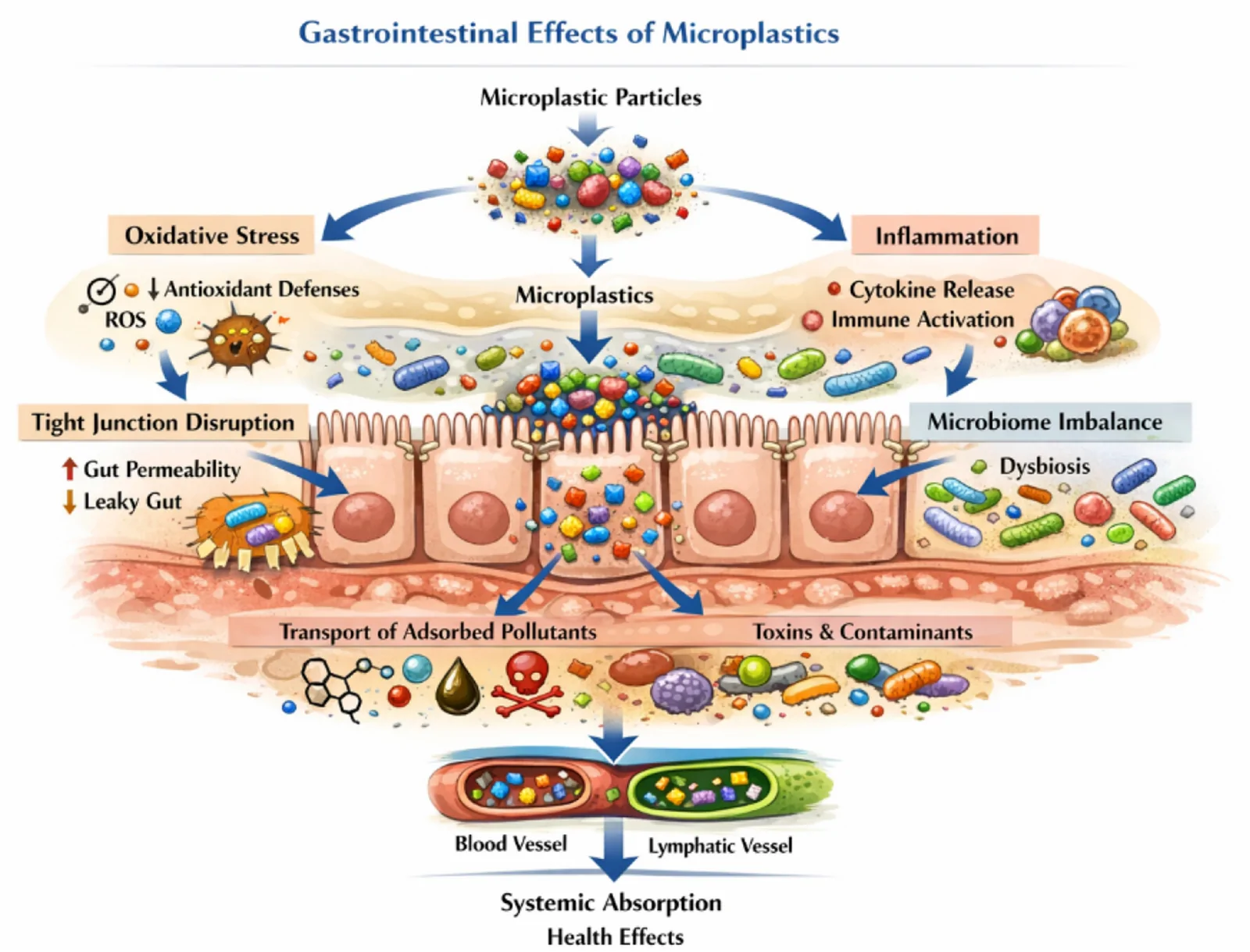
**Gastrointestinal effects of microplastics.** Ingested microplastic particles interact with the intestinal epithelium, triggering oxidative stress, inflammatory responses, and disruption of tight junctions that increase gut permeability. These changes may promote microbiome imbalance, also known as dysbiosis, and facilitate the transport of adsorbed pollutants, toxins, and other contaminants across the intestinal barrier. Subsequent entry into blood and lymphatic vessels may enable systemic distribution and contribute to broader health effects.

MPs ingested through contaminated food, beverages, and food-contact materials are directly introduced into the GI tract, where they may exert a range of adverse biological effects. Increasing evidence indicates that MPs interact with intestinal epithelial surfaces, alter gut barrier integrity, penetrate intestinal tissues, and disrupt host–microbiota homeostasis (Singh et al., 2026). These interactions may contribute to local and systemic toxicity through mechanisms involving inflammation, oxidative stress, immune dysregulation, and metabolic disturbances (Fig. 5).

Recent evidence indicates that disruption of the intestinal epithelial barrier is one of the earliest events following MP exposure. Internalized MPs may impair epithelial homeostasis by inducing oxidative stress, mitochondrial dysfunction, and cytoskeletal remodeling, leading to reduced expression and altered localization of key tight junction proteins, including occludin, claudin-1, and zonula occludens-1 (ZO-1). The resulting increase in intestinal permeability facilitates the translocation of microbial products, endotoxins, and inflammatory mediators into the lamina propria and systemic circulation, thereby activating innate immune signaling and promoting gut–liver axis communication. These interconnected processes provide a mechanistic link between local intestinal injury and systemic inflammatory and metabolic disturbances (Rodríguez-Gutiérrez et al., 2026)**.**

### 5.1 Physical effects

Following ingestion, MPs can mechanically interact with the intestinal epithelium, causing irritation and structural damage to the mucosal surface (Singh et al., 2026). Due to their persistence and resistance to degradation, MPs may accumulate within the GI tract, where prolonged exposure can compromise epithelial integrity and disrupt tight junction proteins. This process contributes to increased intestinal permeability, commonly referred to as the “leaky gut” phenomenon, which facilitates the translocation of luminal toxins, pathogens, and inflammatory mediators into underlying tissues, systemic circulation, and erythrocyte aggregation (Barshtein et al., 2016). Most evidence supporting these mechanisms has been obtained from *in vitro* intestinal cell models and experimental animal studies, with the magnitude of the observed effects varying according to particle size, polymer composition, surface properties, exposure concentration, and duration. Although these studies provide important mechanistic insights, many have employed exposure scenarios that may exceed current estimates of typical human dietary exposure, and their direct clinical relevance remains to be established (Wang et al., 2024a, 2025; Paul et al., 2024).

In addition, MPs and the inflammatory mediators generated in response to exposure can further destabilize key tight junction proteins, including occludin, claudins, and zonula occludens-1 (ZO-1), which are critical for maintaining epithelial barrier homeostasis. Disruption of these junctional complexes enhances paracellular permeability and permits the infiltration of luminal antigens, bacterial components, and other foreign molecules into deeper epithelial and submucosal layers, ultimately promoting local and systemic inflammatory responses (Begley, 1996; Busch et al., 2022). Experimental studies further indicate that these alterations are generally particle size- and dose-dependent, with smaller particles exhibiting greater cellular interaction and translocation across intestinal epithelial barriers than larger particles (Paul et al., 2024).

Studies have reported that MPs can impair mucus secretion, reduce goblet cell abundance, and damage intestinal villi, thereby weakening the protective barrier function of the gut (Chen et al., 2025a; Huang et al., 2023). Such structural alterations may impair nutrient absorption and increase susceptibility to GI disorders and chronic inflammatory conditions. Nevertheless, these pathological changes have been reported predominantly in controlled experimental models, and the persistence and magnitude of intestinal injury following chronic, environmentally relevant dietary exposure in humans remain uncertain (Wang et al., 2024a).

### 5.2 Chemical toxicity and systemic effects

In addition to their physical impacts, MPs may act as carriers for hazardous chemical substances, including plastic additives such as bisphenol A (BPA) and phthalates, as well as adsorbed environmental pollutants such as heavy metals and pesticides. Once internalized, these contaminants may induce cellular dysfunction and injury (Rocha et al., 2025; Kustra et al., 2025). A recent study demonstrated that di(2-ethylhexyl) phthalate (DEHP), a widely used synthetic plasticizer, can co-occur with MPs through intrinsic associations during manufacturing or through environmental interactions after release. Such co-occurrence substantially increases the risk of combined exposure in living organisms. Oral ingestion represents the primary exposure route for both MPs and DEHP, facilitating their passage through the GI tract and subsequent distribution to the enterohepatic system via direct intestinal interactions and systemic circulation (Lan et al., 2026). Furthermore, DEHP has been shown to enhance the cellular uptake of MPs, particularly particles measuring 5 μm, which exhibited a 26% increase in internalization efficiency. Combined exposure to MPs and DEHP produced markedly greater cytotoxic effects than exposure to MPs alone, including a 20% decrease in HepG2 cell viability. Notably, larger particles induced more severe cellular injury, as evidenced by elevated oxidative stress and membrane damage, reflected by a 20% increase in reactive oxygen species (ROS) generation and a 40% increase in lactate dehydrogenase (LDH) release, indicative of compromised membrane integrity (Zhang et al., 2025). Oxidative stress arises from excessive generation of ROS coupled with impairment of endogenous antioxidant defense systems, including superoxide dismutase (SOD), catalase (CAT), and glutathione (GSH) (Chen et al., 2025b; Bocker and Silva, 2025). Persistent oxidative imbalance may damage lipids, proteins, and nucleic acids, ultimately leading to tissue dysfunction and organ injury.

MP-associated toxicity may begin immediately upon oral exposure. Alterations in the oral microbiota have been reported following MP exposure, while epithelial barrier disruption may occur in the esophagus during particle transit (Wen et al., 2024). Within the intestine, accumulated MPs can penetrate epithelial barriers, damage intestinal mucus layers, and impair overall GI tract functionality (Wang et al., 2024a; Paul et al., 2024).

The gut-liver axis represents a critical pathway for systemic MP translocation. Beyond the physical translocation of particles, impairment of intestinal barrier integrity increases portal delivery of bacterial lipopolysaccharide (LPS), microbial metabolites, and inflammatory cytokines to the liver. These mediators activate hepatic Kupffer cells, stellate cells, and resident immune cells through pattern-recognition receptors, particularly Toll-like receptor 4 (TLR4), promoting NF-κB-dependent inflammatory signaling, oxidative injury, and progressive metabolic dysfunction. Thus, disruption of gut barrier function may amplify hepatic injury not only through direct particle accumulation but also through dysregulated gut–liver immune communication. Experimental studies further suggest that, following disruption of the intestinal barrier, MPs can translocate into the circulation and subsequently accumulate in the liver. Evidence indicates that hepatic accumulation of MPs/NPs may induce hepatotoxic effects and impair liver function. Moreover, these effects appear to be size- and dose-dependent, with smaller particles and higher exposure concentrations generally associated with greater hepatic toxicity in experimental models (Beyzaei et al., 2025; Yin et al., 2022). Mechanistic studies further suggest that MPs may disrupt lipid metabolism, induce hepatocyte injury, and activate pyroptosis and ferroptosis signaling pathways (Wang et al., 2024b). It has also been suggested that inhibition of lipase activity following MP accumulation may impair lipid digestion and nutrient utilization (Zheng et al., 2025; Tan et al., 2020). Emerging evidence suggests that MPs may adversely affect pancreatic physiology; however, investigations in this field remain relatively limited. In addition, one study reported that exposure to high concentrations of PET microplastics significantly increased circulating free fatty acid levels in a porcine model. These findings suggest that PET microplastics may disrupt oxidative homeostasis, promote lipotoxic stress, and impair pancreatic exocrine function, thereby revealing a potential mechanistic pathway through which microplastics contribute to metabolic dysfunction (Mierzejewski et al., 2026). In addition, studies have associated MP exposure with pancreatic dysfunction and altered glucose metabolism through disruption of insulin signaling pathways; however, confirmation in human studies is still lacking (Mierzejewski et al., 2026).

Beyond the gastrointestinal system, experimental studies have demonstrated that MPs can accumulate in multiple organs and tissues including the circulatory, nervous, skeletal, and reproductive systems. A recent study demonstrated that MP exposure caused microstructural tissue damage, cellular disorganization, splenic inflammation, elevated malondialdehyde (MDA) and ROS levels, and activation of tumor necrosis factor signaling pathways associated with p38 mitogen-activated protein kinase-mediated cell death.

Therefore, current evidence from *in vitro* and animal studies suggests that chronic MP exposure may promote cellular dysfunction, immune dysregulation, metabolic disturbances, and tissue injury across multiple organ systems. However, direct evidence demonstrating that these mechanisms contribute to chronic disease development in humans remains limited, and further well-designed epidemiological and clinical studies are required to establish their clinical relevance.

### 5.3 Inflammation and immune responses

Exposure to MPs has been increasingly linked to intestinal inflammation and dysregulated immune responses. Persistent accumulation of MPs within the GI tract may promote immune cell infiltration and sustain chronic low-grade inflammation (Covello et al., 2024). MPs can be recognized by macrophages as foreign particulate matter through Fc receptor-mediated interactions, thereby activating multiple intracellular signaling pathways (Bianchi et al., 2025). These responses include autophagy, in which cells attempt to degrade or sequester internalized particles; metabolic reprogramming involving activation of the tricarboxylic acid (TCA) cycle; and genotoxic effects associated with damage to cellular genetic material. Together, these processes enhance the generation of ROS, including superoxide anions, hydrogen peroxide (H_2_O_2_), and highly reactive hydroxyl radicals. Excessive ROS production subsequently induces oxidative stress, stimulates pro-inflammatory cytokine release, and amplifies immune signaling cascades, ultimately contributing to intestinal inflammation and immune dysfunction (Singh et al., 2026).

MP-induced barrier disruption may facilitate the passage of luminal antigens, bacterial components, toxins, and other foreign molecules into deeper epithelial and submucosal layers, subsequently activating TLRs, particularly TLR4 and TLR9, expressed on intestinal epithelial and immune cells, thereby initiating downstream inflammatory signaling cascades (Adler et al., 2024). TLR activation stimulates intracellular adaptor pathways, including myeloid differentiation primary response 88 (MyD88) and mitogen-activated protein kinase (MAPK) signaling pathways, leading to the activation of pro-inflammatory transcription factors such as activator protein-1 (AP-1) and interferon regulatory factor-5 (IRF-5). Notably, MAPK signaling plays a pivotal role in regulating multiple cellular processes, including proliferation, differentiation, apoptosis, and cellular stress responses (K et al., 2023). These signaling events promote the expression and release of pro-inflammatory cytokines, including tumor necrosis factor-α (TNF-α), interleukin-6 (IL-6), and interleukin-1β (IL-1β). Sustained cytokine production exacerbates intestinal inflammation, compromises epithelial and vascular barrier integrity, and may further amplify systemic immune activation and inflammatory responses. In addition to TLR signaling, increasing evidence indicates that MPs activate the NLRP3 inflammasome, promoting caspase-1 activation and maturation of IL-1β and IL-18. Persistent activation of these innate immune pathways may sustain chronic low-grade intestinal inflammation, impair epithelial repair, and further increase intestinal permeability, thereby establishing a self-perpetuating cycle of barrier dysfunction and immune activation (Samanta et al., 2025).

Several studies have specifically investigated the contribution of MPs to GI diseases and related pathological conditions. Among these, PS-MPs have been shown to induce chronic colitis following dietary exposure, highlighting their potential significance as an emerging global public health concern (Krasucka et al., 2022). A recent study further demonstrated that cyanidin-3-glucoside (C3G) may alleviate PS-induced gut dysbiosis and colonic inflammation, suggesting that modulation of gut microbiota and inflammatory pathways could represent a potential therapeutic strategy for MP-associated intestinal disorders (Wu et al., 2026). In experimental murine models, PS-MP exposure significantly increased the expression of pro-inflammatory cytokines, including TNF-α, IL-6, and IL-1β, leading to immune cell activation, migration, and infiltration within colonic tissues. Elevated inflammatory signaling disrupts tight junction integrity between intestinal epithelial cells, reduces goblet cell populations, and enhances intestinal permeability. Consequently, luminal contents and microbial products more readily penetrate the submucosal layer, perpetuating a self-amplifying inflammatory cycle that exacerbates tissue injury and epithelial dysfunction. PS exposure has been associated with hepatic lipid accumulation, disruption of cellular energy homeostasis, and induction of several cell death mechanisms, including pyroptosis, apoptosis, and ferroptosis (Tan et al., 2020).

### 5.4 Microbiome disruption

The gut microbiota plays a critical role in maintaining intestinal homeostasis, immune regulation, and metabolic function. Accumulating evidence suggests that MPs can significantly alter the composition and diversity of gut microbial communities, thereby contributing to dysbiosis (Sinha et al., 2025). Across experimental studies, the most consistently reported microbiota alterations include reduced microbial diversity, enrichment of opportunistic or pathogenic bacteria, and disruption of beneficial commensal populations, although the magnitude of these changes varies according to particle characteristics, exposure conditions, and host species. Changes in microbial populations may favor the proliferation of opportunistic and pathogenic bacteria, including *Escherichia coli* and Proteus species, which produce metabolites such as trimethylamine that can damage intestinal epithelial cells and promote inflammatory responses (Lan et al., 2026).

This dysbiosis promotes the release of pro-inflammatory mediators, including IL-1α/β, TNF-α, and interferon-gamma (IFN-γ), through the activation of specific receptors within the intestinal barrier. One study investigated the effects of food chain–transferred NPs on the gut–liver axis in mice (Qu et al., 2026). The findings demonstrated that NP exposure induced gut microbiota dysbiosis, disrupted metabolic homeostasis, and altered hepatic gene expression. Notably, upregulation of the *Cyp26a1* gene was identified as a key mechanism underlying NP-induced hepatotoxicity through disruption of retinoic acid metabolism and the development of hypervitaminosis A. The study further revealed significant associations between alterations in gut microbial composition and bile acid metabolism, emphasizing the critical role of the gut–liver axis in mediating NP-induced metabolic and hepatic dysfunction. Recent advances in multi-omics technologies have provided deeper mechanistic insights into MP-induced gut microbiota dysbiosis. Integrated metagenomic, metabolomic, and transcriptomic analyses demonstrate that MP exposure not only alters microbial composition but also disrupts microbial functional pathways involved in bile acid metabolism, short-chain fatty acid biosynthesis, amino acid metabolism, and lipid homeostasis. These metabolic alterations are closely associated with impaired intestinal barrier function, dysregulated immune responses, and altered hepatic metabolic signaling through the gut–liver axis. Furthermore, transcriptomic analyses have identified differential expression of genes involved in epithelial barrier maintenance, inflammatory signaling, xenobiotic metabolism, and oxidative stress responses, highlighting complex host–microbiota interactions that cannot be captured by microbial composition alone (Chen et al., 2025a; Jin et al., 2019). Therefore, these multi-omics approaches provide a more comprehensive understanding of the molecular mechanisms linking MP-induced dysbiosis to gastrointestinal and systemic metabolic dysfunction. Increasing evidence further suggests that MPs/NPs impair host physiological homeostasis by disrupting tight junction proteins and modulating the activity of key effector cells (Ghosh and Gorain, 2025). MP-induced dysbiosis may further aggravate intestinal barrier dysfunction, inflammation, and oxidative stress, establishing a detrimental feedback loop between microbial imbalance and epithelial injury.

In parallel, MP-induced dysbiosis of the intestinal microbiota can profoundly perturb host immune homeostasis, thereby potentially contributing to chronic disease development, enhanced susceptibility to pathogenic infection, and altered microbial gene composition and expression profiles. Previous studies have consistently demonstrated that MP exposure induces marked intestinal pathology, predominantly characterized by disruption of the intestinal mucosal barrier (Wang et al., 2024b; Fackelmann and Sommer, 2019). This barrier dysfunction may subsequently permit the systemic dissemination of intestinal endotoxins, inflammatory mediators, and microbiota-derived metabolites through translocation into the circulatory system. Nevertheless, despite growing evidence from experimental animal models, mechanistic understanding of these processes in humans remains limited, underscoring the need for clinically relevant translational investigations.

Particle size has emerged as a critical determinant of MP toxicity, particularly in relation to gut microbiota dysbiosis and intestinal barrier dysfunction. Larger particles, approximately 50 nm in size, have been reported to induce greater oxidative stress compared with smaller particles, approximately 45 nm in size, whereas smaller particles appear to exert more pronounced effects on intestinal microbial homeostasis. An *in vivo* study reported that oral exposure to MPs significantly altered the composition and diversity of the intestinal microbiota (Lu et al., 2018).

MP-induced dysbiosis may compromise the integrity and thickness of the intestinal mucus layer, thereby facilitating abnormal mucus penetration and enhanced bacterial colonization of epithelial surfaces. In addition, disruption of the mucosal barrier may increase direct interactions between MPs and intestinal epithelial cells, resulting in epithelial injury, barrier dysfunction, and heightened susceptibility to intestinal inflammation. However, several of these microbial and immunometabolic alterations, including dysbiosis, cell dysfunction, and inflammatory activation, have also been reported in response to other dietary and environmental stressors. Therefore, although current experimental evidence supports a role for MPs/NPs in perturbing the gut microbiota and gut-liver axis, the specificity of these responses to MPs/NPs and their contribution relative to other environmental exposures remain to be fully elucidated.

Despite growing recognition of these effects, effective strategies to restore microbiota balance and mitigate MP-induced intestinal inflammation remain limited and require further investigation.

Table 1 summarizes key experimental studies discussed in this section, highlighting model systems, particle characteristics, exposure conditions, and their relevance to human gastrointestinal health.

**Table 1:** Key experimental studies on microplastic and nanoplastic gastrointestinal toxicity and their relevance to human health.

Study	Model	Particle Type/Size and Polymer	Dose/Concentration and Duration	Main Endpoints and Outcomes	Potential translational relevance
(Lan et al., 2026)	*In vivo*, mice (C57/BL6J)	Polystyrene microplastics (PS-MPs) ± DEHP (plasticizer)	Not specified dose; 8 weeks oral exposure	Enhanced enterohepatic inflammation and oxidative stress; intestinal microbial disturbance; effects transferable via fecal microbiota transplantation	Supports a potential role of gut microbiota as a mediator of combined MP/plasticizer toxicity along the gut–liver axis, with hypothesis-generating relevance for human co-exposure scenarios.
(Mierzejewski et al., 2026)	*In vivo*, pig (proteomic/LC-MS/MS)	PET microplastics	Low dose 0.1 g/day vs. high dose 1 g/day; 4 weeks	Dose-dependent proteomic changes (7 vs. 17 proteins altered); disrupted fatty acid biosynthesis (FASN, KAS) and lipid peroxidation (CBR1); increased free fatty acids; lipotoxic stress; impaired pancreatic exocrine function	Because pigs share physiological similarities with humans, this model provides potential translational relevance and identifies a hypothesis-generating metabolic/pancreatic disruption pathway related to dietary MP exposure.
(Zhang et al., 2025)	*In vitro*, human HepG2 cells (3D spheroids)	PS-MPs (100 nm, 1 μm, 5 μm) ± DEHP	100 μg/mL, DEHP: 25.6 μM, short-term exposure	100 nm particles showed highest internalization; DEHP increased 5 μm particle internalization by 26%; co-exposure reduced cell viability by 20%; ROS increased 20%; LDH release increased 40%	Provides a human hepatic cell model supporting biological plausibility that plasticizer co-exposure may potentiate MP internalization and cytotoxicity, with potential relevance to gut–liver translocation.
(Wu et al., 2025)	*In vitro*, human THP-1 cells	Micro/nanoplastics (MNPs) released from photoaged PLA/PBAT biodegradable film	Dose-dependent; EC_50_ = 243 mg/L (ultrafiltration-derived MNPs)	Photoaging increased MNP release 18-28× under GI-simulated conditions; dose-dependent cytotoxicity; toxicity comparable to or greater than conventional plastic MNPs	Highlights biodegradable plastic packaging as an underrecognized GI-relevant MNP source and suggests that toxicity may not necessarily be lower than that of conventional plastics
(Qu et al., 2026)	*In vivo*, mice (fed mealworms exposed to PS-NPs)	Polystyrene nanoplastics (PS-NPs), 80 nm; food chain-transferred (FCT-NPs) via yellow mealworms	Mealworms reared on 80 nm PS-NP-spiked agar diet; 24 days	Reduced gut microbiota alpha diversity (Chao1, observed species, Faith's PD, Pielou's evenness); altered beta diversity/community structure (NMDS, PCoA); *Cyp17a1* upregulated ∼11.5-fold and *Cyp26a1* upregulated ∼52.5-fold (both p < 0.0001); disrupted retinoic acid and bile acid metabolism; hepatic transcriptomic/metabolomic changes	Supports *CYP26A1*-mediated retinoic acid dysregulation as a potential gut–liver axis mechanism and provides hypothesis-generating evidence that trophic transfer of nanoplastics may contribute to systemic hepatotoxicity relevant to animal-derived dietary exposure.
(Lu et al., 2018)	*In vivo* (mice)	MPs; larger (∼50 nm) vs. smaller (∼45 nm) particles	Oral exposure; duration not specified	Larger particles induced greater oxidative stress; smaller particles more strongly altered gut microbiota composition/diversity	Supports the biological plausibility of particle size-dependent divergent effects on oxidative stress and microbiota composition, with potential relevance for human gastrointestinal exposure.

## 6 Biological plausibility representative preclinical studies

### 6.1 In vitro studies-cell culture models

Several *in vitro* studies have demonstrated that MPs and NPs exert cytotoxic, genotoxic, inflammatory, and metabolic effects in mammalian cells, although the magnitude and nature of these responses vary considerably depending on particle size, polymer type, concentration, exposure duration, and cell type, limiting direct comparisons across studies. These findings support mechanistic hypotheses regarding potential disease pathogenesis, including cancer, but do not establish causal relationships in humans (Bailey and Hofseth, 2026).

The biological effects of cryo-milled particles derived from single-use plastic products, including plastic forks and cups, were evaluated in eight mammalian cell lines. Exposure to MPs at a concentration of 100 μg/mL did not significantly affect cell viability in Caco-2, HEK001, MRC-5, or T47D cells. However, HMEC-1 endothelial cells exhibited significantly reduced viability following exposure to fork-derived particles at concentrations ranging from 10 to 100 μg/mL. Likewise, HMC-3 and HepaRG cells demonstrated decreased viability after treatment with cup-derived particles at 100 μg/mL. Interestingly, cup-derived MPs promoted HMEC-1 cell proliferation across concentrations ranging from 0.1 to 100 μg/mL. These cell type-specific responses suggest that MP toxicity is not uniform across tissues and highlight the importance of considering cellular susceptibility when interpreting experimental findings. Nevertheless, the relatively high exposure concentrations and acute experimental conditions may not accurately reflect typical human dietary exposure (Janiga-MacNelly et al., 2025).

Accumulating evidence further suggests that cellular exposure to micro/nanoplastics (MNPs) induces profound molecular, metabolic, and biological alterations that may ultimately contribute to pathological outcomes. However, most available evidence is derived from experimental cell models, and the relevance of these molecular changes to human carcinogenesis remains uncertain. Considering the chronic and lifelong human exposure to progressively increasing levels of environmental MNPs, their involvement in carcinogenesis and tumor progression has become an emerging area of concern (Cirillo et al., 2026). Preclinical studies suggest that MNPs may promote cancer initiation and progression through multiple interconnected mechanisms, including DNA damage, chronic inflammation, metabolic reprogramming, immune dysregulation, and endocrine disruption (Deng et al., 2025). Exposure of HT-29 and HCT116 colorectal cancer cell lines to PE-MPs (10 μg/mL) for up to 72 h significantly enhanced cell proliferation, inflammatory signaling, angiogenic activity, and invasive potential. PE exposure significantly enhanced cell proliferation (*p* < 0.001), inflammatory signaling, angiogenic activity, and invasive potential.

Moreover, PE-treated cells displayed increased cellular bioenergetics, characterized by enhanced glycolysis and mitochondrial respiration (*p* < 0.05). At the molecular level, PE exposure upregulated the expression of Sirtuin 1 **(**SIRT1) and sodium-glucose cotransporter 2 (SGLT2) proteins (*p* < 0.05), suggesting a shift toward an oncogenic metabolic phenotype (Donisi et al., 2026). Importantly, pharmacological inhibition of SGLT2 (i2) attenuated the pro-tumorigenic effects induced by PE exposure by reducing cell proliferation, inflammatory responses, glycolytic activity, and mitochondrial respiration (*p* < 0.001). In parallel, iSGLT2 treatment promoted lipid peroxidation and ferroptotic cell death (*p* < 0.001), highlighting a potential therapeutic strategy targeting the metabolic vulnerabilities of colorectal cancer cells. Similarly, treatment with PE, γ-butyrobetaine (γBB), and canagliflozin (Cnt) modulated onco-metabolic alterations and induced ferroptosis (Donisi et al., 2026). While these findings provide mechanistic evidence that MPs can influence cancer-related signaling pathways, they were obtained exclusively in established cancer cell lines and should not be interpreted as evidence that microplastic exposure initiates or promotes colorectal cancer in humans.

One study reported that ultraviolet photoaging of biodegradable PLA/PBAT plastic, composed of polylactic acid and poly(butylene adipate-co-terephthalate), markedly increased the release of MNPs, particularly under GI conditions, where mass loss was up to 28-fold higher than in unaged plastics. The released MNPs induced dose-dependent cytotoxicity in THP-1 cells, suggesting that biodegradable plastic-derived MNPs may exhibit toxicity comparable to or greater than that of conventional plastic particles (Wu et al., 2025). Furthermore, it has been demonstrated that PET- and PVC-NPs induced greater cytotoxicity in HepG2 cells than PS-NPs, primarily through oxidative stress, mitochondrial apoptosis, and suppression of DNA repair- and lipid metabolism-related genes. These findings suggest that NP toxicity is strongly influenced by polymer composition and physicochemical properties (Ma et al., 2024). This observation highlights that results obtained using a single polymer type cannot be generalized to all MPs or NPs, emphasizing the need for standardized testing across environmentally relevant polymer mixtures.

In addition, it has been reported that exposure to micro-sized polyethylene particles reduced cell viability and increased oxidative stress, particularly mitochondrial superoxide production, in Caco-2 and HT-29 intestinal cells, highlighting the potential cytotoxic effects of PE-MPs on the GI epithelium (Herrala et al., 2023). Another study indicated that perfluorooctane sulfonate (PFOS) significantly enhanced the uptake of polystyrene nanoparticles by Caco-2 intestinal cells by more than 30%, thereby altering particle surface properties and increasing oxidative stress, membrane damage, and mitochondrial dysfunction, highlighting the potential combined toxicity of microplastics and persistent organic pollutants (Liu et al., 2022). Therefore, these *in vitro* studies consistently demonstrate that MPs and NPs can trigger cellular dysfunction pathways and metabolic alterations. However, considerable methodological heterogeneity, including differences in particle characteristics, exposure concentrations, cell models, and experimental endpoints, limits cross-study comparability and underscores the need for standardized *in vitro* protocols.

### 6.2 Animal studies in mouse models

Animal studies provide important preclinical evidence for understanding the biological effects of MPs and NPs under controlled exposure conditions. However, differences in exposure doses, particle characteristics, administration routes, and species-specific physiology limit the direct extrapolation of these findings to human health. Evidence from animal models indicates that MPs and NPs exert multisystem toxic effects, particularly within the GI, hepatic, metabolic, and immune systems. Experimental studies consistently demonstrate that oral exposure to MPs/NPs disrupts intestinal barrier integrity, promotes chronic inflammation, alters host metabolism, and induces gut microbiota dysbiosis, providing mechanistic evidence that these particles can affect multiple physiological systems under experimental conditions.

In healthy murine colon models, MPs have been shown to impair intestinal barrier function, promote inflammatory responses, increase immune cell infiltration, and alter epithelial homeostasis. Furthermore, MP exposure disrupts epithelial homeostasis by altering cellular proliferation, apoptosis, differentiation, tight junction integrity, glycocalyx composition, membrane transport processes, intracellular signaling pathways, and intestinal metabolomic profiles, while also reshaping gut microbial communities. Importantly, in both acute and chronic experimental colitis models, MP ingestion exacerbated intestinal inflammation and disease severity, suggesting that MPs may contribute to IBD-like pathology in experimental animals. However, these findings have not yet been confirmed in humans, and differences between murine models and human GI physiology should be considered when interpreting their clinical relevance (Zolotova et al., 2025).

Available evidence further demonstrates that co-exposure to PS-NPs and glyphosate for 18 days markedly aggravated hepatotoxicity in mice through enhanced oxidative stress, neutrophil infiltration, neutrophil extracellular trap (NET) formation, and inflammatory pyroptosis. Mechanistically, the combined exposure activated the TLR4/NF-κB/NLRP3 inflammasome signaling pathway, indicating that NET-mediated inflammasome activation plays a pivotal role in PS-NP- and glyphosate-induced liver injury (Zeng et al., 2025). These findings also highlight that co-exposure to environmental contaminants may substantially modify MP toxicity, an important consideration that is often overlooked when comparing experimental studies. Similarly, a recent study demonstrated that chronic exposure to PS-NPs at 1000 μg/L for 12 weeks induced obesity, hepatic steatosis, and inflammatory responses in mice, accompanied by substantial alterations in lipid metabolites and cytochrome P450-related genes (Chen et al., 2025b). Integrated metabolomic and transcriptomic analyses revealed that dysregulated phosphatidylcholine metabolism and arachidonic acid-associated signaling pathways may represent key mechanisms underlying NP-induced hepatic lipid metabolic dysfunction (Chen et al., 2025b). Although these mechanistic data strengthen the biological plausibility of MP-induced metabolic disorders, the exposure conditions used in animal experiments frequently exceed estimated human dietary exposure levels, limiting quantitative risk extrapolation.

In addition, one study reported that sub-chronic exposure to PET-NPs significantly altered both gut and lung microbiota composition in mice. These changes were characterized by increased abundance of pro-inflammatory Gram-negative bacteria, including *Veillonella*, *Prevotella*, and *Pseudomonas*, along with reduced *Lactobacillus* populations and perturbations in microbial metabolic pathways, highlighting the capacity of NPs to disrupt the gut–lung microbial axis (Kaluç et al., 2024). Moreover, another study investigated the systemic distribution and metabolomic consequences of oral exposure to polystyrene and mixed MPs (5 μm), including PS, PE, and poly(lactic-co-glycolic acid), in mice. The findings demonstrated that ingested MPs can translocate across the intestinal barrier, enter systemic circulation, and accumulate in distant organs, including the brain, liver, and kidneys. Importantly, MP exposure induced dose- and plastic type-dependent metabolomic alterations in the colon, liver, and brain, providing evidence that MPs may exert widespread systemic effects extending beyond the gastrointestinal tract (Garcia et al., 2023).

Therefore, animal studies consistently demonstrate that orally administered MPs and NPs can disrupt intestinal integrity, alter microbial communities, impair metabolic homeostasis, and affect distant organs under controlled experimental conditions. Nevertheless, substantial variability in particle type, size, dose, exposure duration, and animal models complicate comparisons among studies. Moreover, because these findings are derived exclusively from preclinical models, their relevance to human health requires confirmation through well-designed epidemiological and clinical investigations.

### 6.3 Human evidence

Human evidence regarding the biological and pathological effects of MPs remains scarce and is currently limited to a small number of exploratory and proof-of-concept investigations. Nevertheless, emerging studies provide preliminary evidence supporting the systemic translocation and tissue accumulation of MPs in humans.

Current evidence indicates that dietary intake represents one of the principal routes of human exposure to MPs, with drinking water, seafood, table salt, fruits, vegetables, and food packaging identified as major contributors. Although estimates of dietary intake vary considerably because of differences in analytical methodologies, particle size detection limits, and contamination control, available studies consistently suggest that humans are chronically exposed to MPs at relatively low environmental concentrations. Importantly, these estimated exposure levels are substantially lower than the doses commonly employed in in vitro and animal studies, which are often designed to investigate toxicological mechanisms rather than to replicate realistic human exposure scenarios. Consequently, caution is warranted when extrapolating experimental findings to human health risk, highlighting the need for standardized exposure assessment methods and studies employing environmentally relevant exposure levels.

Notably, one of the first proof-of-concept human studies examined MP accumulation in hepatic tissue. Using a combination of Raman spectroscopy and fluorescence microscopy, the authors identified six distinct MP polymers measuring 4–30 μm exclusively in cirrhotic liver tissues, whereas liver, kidney, and spleen samples obtained from individuals without underlying liver disease showed no detectable MPs. These findings provide the first direct evidence of MP deposition in human cirrhotic liver tissue and suggest a possible association between chronic liver disease and MP accumulation. However, because of the small sample size and observational design of this study, it remains unclear whether MP accumulation contributes to disease progression or is a consequence of pathological changes associated with cirrhosis, such as impaired intestinal barrier function and reduced hepatic clearance. Therefore, the proposed role of MPs in hepatic inflammation, fibrogenesis, and liver dysfunction remains biologically plausible but unproven in humans (Horvatits et al., 2022).

Emerging human studies also suggest that the gut microbiome may influence the environmental fate of MPs within the gastrointestinal tract. A recent study isolated bacterial species from fecal samples of healthy individuals that were capable of degrading LDPE and PP, providing preliminary evidence that human gut-associated bacteria may participate in MP biodegradation and biotransformation. These findings raise the possibility that microbial metabolism may generate smaller secondary MPs, NPs, or intermediate degradation products capable of influencing intestinal homeostasis, microbial ecology, and host immune responses. However, these observations are currently based on limited exploratory data, and their physiological relevance and implications for human health require further investigation (Jang et al., 2024).

Overall, the available human evidence supports the presence of MPs in human tissues and suggests potential interactions with host biological systems. However, the current evidence base is limited by small sample sizes, heterogeneous analytical methodologies, and predominantly observational study designs. Large, well-characterized epidemiological studies and longitudinal clinical investigations are therefore needed to determine exposure–response relationships, identify susceptible populations, and establish whether MPs contribute directly to human disease (Table 2).

**Table 2:** Representative preclinical and human studies on MP/NP biological effects.

Study	Study type/model	Particle characteristics	Exposure conditions	Principal findings	Major limitations
(Janiga-MacNelly et al., 2025)	In vitro – 8 mammalian cell lines (Caco-2, HEK001, MRC-5, T47D, HMEC-1, HMC-3, HepaRG)	Cryo-milled MPs from single-use plastic forks and cups	0.1–100 μg/mL	Cell type–specific effects: reduced viability in HMEC-1 (fork-derived, 10–100 μg/mL) and HMC-3/HepaRG (cup-derived, 100 μg/mL); cup-derived MPs promoted HMEC-1 proliferation	High, acute exposure concentrations; non-uniform responses across cell types limit generalization
(Donisi et al., 2026)	In vitro – HT-29, HCT116 (colorectal cancer cell lines)	PE microplastics	10 μg/mL, up to 72 h	↑ Proliferation, inflammatory signaling, angiogenesis, invasion; ↑ glycolysis/mitochondrial respiration; ↑ SIRT1/SGLT2; SGLT2 inhibition reversed pro-tumorigenic effects and induced ferroptosis	Restricted to established cancer cell lines; not evidence of *in vivo* carcinogenesis
(Wu et al., 2025)	In vitro – THP-1 cells	MNPs from UV-photoaged biodegradable PLA/PBAT plastics	Dose-dependent exposure	Dose-dependent cytotoxicity; photoaging markedly increased MNP release	Biodegradable plastics may not be inherently safer; single cell line
(Ma et al., 2024)	In vitro – HepG2 cells	PET-, PVC-, and PS-NPs	Experimental exposure	PET- and PVC-NPs more cytotoxic than PS-NPs via oxidative stress, mitochondrial apoptosis, suppressed DNA repair/lipid metabolism genes	Polymer-specific results may not be generalizable to all MPs/NPs
(Herrala et al., 2023)	In vitro – Caco-2, HT-29 intestinal cells	Micro-sized polyethylene particles	Experimental exposure	↓ Cell viability; ↑ oxidative stress (mitochondrial superoxide)	Single polymer type; intestinal cell lines only
(Liu et al., 2022)	In vitro – Caco-2 cells	PS-NPs + perfluorooctane sulfonate (PFOS)	Co-exposure	PFOS ↑ PS-NP uptake by >30%; ↑ oxidative stress, membrane damage, mitochondrial dysfunction	Co-exposure model; limited to one polymer/pollutant combination
(Zolotova et al., 2025)	Animal – murine colon (healthy and colitis models)	MPs, oral administration	Acute and chronic exposure	Impaired intestinal barrier, ↑ inflammation, immune infiltration, dysbiosis; exacerbated colitis severity	Species-specific physiology; not yet confirmed in humans
(Zeng et al., 2025)	Animal – mice	PS-NPs	10 nM + glyphosate, 18 days	Aggravated hepatotoxicity via oxidative stress, NETs, pyroptosis; TLR4/NF-κB/NLRP3 activation	Combined-exposure design; human dose relevance unclear
(Chen et al., 2025b)	Animal – mice	PS-NPs	1000 μg/L, continuous, 12 weeks	Obesity, hepatic steatosis, inflammation; dysregulated phosphatidylcholine and arachidonic acid metabolism	Exposure doses exceed estimated human dietary intake
(Kaluç et al., 2024)	Animal – mice	PET-NPs	Sub-chronic exposure	Altered gut/lung microbiota: ↑ pro-inflammatory Gram-negative bacteria (Veillonella, Prevotella, Pseudomonas); ↓ Lactobacillus	Mechanistic and human relevance unclear
(Garcia et al., 2023)	Animal – mice	PS, PE, PLGA MPs	5 μm, oral	Translocation across intestinal barrier; accumulation in brain, liver, kidney; dose- and polymer-dependent metabolomic changes	Preclinical evidence only; the extent of human translocation remains unconfirmed
(Horvatits et al., 2022)	Human – cirrhotic vs. non-cirrhotic liver, kidney, spleen tissue	6 MP polymers, 4–30 μm	Liver tissue analysis (Raman spectroscopy/fluorescence microscopy)	MPs detected exclusively in cirrhotic liver tissue; absent in non-diseased tissue	Small sample size; observational design; causality, i.e., cause versus consequence, remains unclear
(Jang et al., 2024)	Human – fecal bacterial isolates	LDPE, PP (environmental MPs)	Ex vivo bacterial culture from healthy individuals	Gut bacteria capable of degrading LDPE/PP; potential generation of secondary MPs/NPs	Exploratory data; physiological/health relevance not established

## 7 Risk assessment challenges

A major source of uncertainty in risk assessment is the analytical variability associated with MP detection and quantification. Although Raman spectroscopy, FTIR spectroscopy, laser direct infrared (LDIR) imaging, and pyrolysis-gas chromatography/mass spectrometry (Py-GC/MS) are among the most widely used analytical techniques, each possesses distinct advantages and limitations regarding particle size detection limits, polymer identification, quantitative accuracy, sample throughput, and compatibility with complex biological matrices. Furthermore, inadequate contamination control, differences in sample collection and digestion protocols, and inconsistent quality assurance procedures contribute to substantial variability among studies. Consequently, reported concentrations and polymer profiles are often difficult to compare directly, limiting accurate exposure assessment and reliable health risk characterization.

Another critical limitation is the absence of well-defined safe exposure thresholds for humans and non-human biota, including wildlife, hindering accurate risk characterization and ecological hazard assessment. Current evidence regarding the long-term health effects of chronic low-dose exposure remains insufficient, particularly with respect to gastrointestinal, metabolic, immunological, and systemic toxicity (Covello et al., 2024). In addition, the biological behavior and toxic potential of MPs are highly influenced by their physicochemical characteristics, including particle size, shape, polymer type, surface charge, and chemical additives or adsorbed pollutants. Polymer composition influences particle stability, chemical reactivity, and additive release, whereas particle morphology and size determine cellular uptake, tissue distribution, and bioavailability. Surface aging and environmental weathering can modify particle surface chemistry, increase surface roughness, and alter interactions with biological systems, while changes in surface charge affect cellular membrane interactions and particle internalization. Moreover, MPs can adsorb environmental contaminants, such as persistent organic pollutants and heavy metals, potentially modifying their transport, bioavailability, and toxicological effects (Zhou et al., 2026; Tan et al., 2026). Smaller particles, especially NPs, may exhibit greater cellular penetration and bioavailability, whereas irregularly shaped particles may induce enhanced mechanical and inflammatory damage. However, these relationships remain insufficiently characterized and require further systematic investigation.

Risk evaluation is further complicated by the ability of MPs to interact with environmental contaminants, microorganisms, and endogenous biomolecules, thereby altering their transport, bioaccumulation, and toxicological properties. Moreover, differences in exposure routes, exposure duration, and host susceptibility factors such as age, diet, microbiota composition, and pre-existing disease conditions may significantly influence toxic outcomes. Therefore, these challenges highlight the urgent need for harmonized analytical methodologies, long-term exposure studies, and mechanistic investigations to establish reliable health risk assessment frameworks for MPs and NPs.

## 8 Mitigation in food production

Mitigating microplastic contamination within food production systems requires integrated strategies spanning agricultural practices, food processing, and packaging technologies. Reducing the use of conventional plastics in agriculture, including plastic mulching films, irrigation materials, fertilizers coated with polymeric substances, and greenhouse coverings, represents a critical step toward minimizing microplastic release. The adoption of biodegradable or sustainable alternatives, together with improved waste management and recycling practices, may substantially decrease plastic accumulation in agricultural soils and water systems, although their comparative environmental and safety benefits should not be assumed a priori and require confirmation through degradation studies under realistic field conditions (Steinmetz et al., 2016).

Advances in food processing technologies are also essential for limiting microplastic contamination during manufacturing, transportation, and storage (Matondo et al., 2026). Improved filtration systems, contamination monitoring, hygienic processing conditions, and stricter quality control measures can reduce the introduction of MPs into food products. In addition, minimizing plastic contact surfaces throughout food production chains may further decrease particle migration into consumable materials (Snekkevik et al., 2024).

The development of safer packaging alternatives constitutes another important mitigation approach. Replacing conventional plastic packaging with biodegradable, recyclable, or bio-based materials may reduce the release of MPs and harmful plastic-associated chemicals into food products; however, such materials should not be presumed inherently safer, as their comparative safety is conditional on migration testing, realistic use conditions, particle characterization, and life-cycle assessment, and some biodegradable polymers may generate their own micro- and nanoplastic fragments or novel degradation by-products under real-world conditions. Furthermore, the design of sustainable food-contact materials with enhanced stability and lower degradation potential could contribute significantly to reducing chronic dietary exposure to MPs and associated contaminants, provided these properties are verified through rigorous, standardized testing rather than assumed on the basis of material origin alone (Nuojua et al., 2024).

In parallel, regulatory agencies and international organizations have increasingly recognized MPs as an emerging food safety concern. Although no harmonized regulatory limits for MPs in food currently exist, organizations such as the World Health Organization (WHO), the Food and Agriculture Organization (FAO), the European Food Safety Authority (EFSA), and the European Commission have emphasized the need for standardized analytical methodologies, harmonized monitoring programs, improved exposure assessment, and comprehensive human health risk evaluation. Within the food industry, implementation of good manufacturing practices (GMP), hazard analysis and critical control point (HACCP)-based quality management, routine contamination monitoring, and the replacement of high-wear plastic equipment with more durable alternatives may reduce secondary MP contamination during food processing (Puteri et al., 2025). Emerging technologies, including advanced membrane filtration, nanofiltration, electrocoagulation, magnetic separation, and biodegradable food-contact materials, also show promise for reducing MP contamination throughout the food production chain, although further validation under real-world industrial conditions is required. In particular, the comparative safety and performance of biodegradable materials should be evaluated on a case-by-case basis using migration and life-cycle data rather than treated as default safer options (Acarer, 2023; Ahmed et al., 2026).

## 9 Limitations of current toxicological studies

Current toxicological evidence has substantially advanced our understanding of the biological effects of MPs and NPs; however, several important limitations should be considered when interpreting these findings. Many *in vitro* and animal studies employ exposure concentrations that exceed estimated human environmental and dietary exposure levels, primarily to investigate potential toxicological mechanisms. In addition, most experimental studies use pristine, commercially manufactured particles with uniform size, shape, and polymer composition, whereas human exposure involves heterogeneous mixtures of environmentally weathered particles carrying adsorbed contaminants and undergoing aging processes. Experimental exposure protocols are also frequently acute or short-term and often rely on simplified exposure routes that may not accurately reflect chronic dietary exposure in humans. Therefore, these methodological limitations reduce the direct translation of experimental findings to human health risk and underscore the need for studies employing environmentally relevant particles, realistic exposure scenarios, and standardized experimental designs.

## 10 Research gaps and future directions

Despite increasing evidence indicating potential adverse health effects of MPs and NPs, several critical knowledge gaps remain unresolved. Current understanding is largely based on *in vitro* experiments and short-term animal studies, whereas long-term, well-controlled human investigations—particularly those addressing GI exposure, bioaccumulation, and clinical outcomes—remain extremely limited. Consequently, comprehensive epidemiological and clinical cohort studies are urgently needed to clarify chronic exposure effects, systemic distribution patterns, and the potential contribution of MPs/NPs to gastrointestinal, metabolic, and other systemic diseases in humans. Importantly, human evidence to date is still scarce and largely exploratory, underscoring the need for rigorous translational research to bridge the gap between experimental findings and real-world health outcomes.

Another key research priority is the characterization of interactions between MPs/NPs and the gut microbiome. Although emerging evidence suggests that MPs can induce microbial dysbiosis, alter metabolic pathways, and impair intestinal barrier integrity, the underlying mechanistic links within the host-microbiota-plastic axis remain insufficiently understood. Future studies integrating multi-omics approaches, including metagenomics, metabolomics, transcriptomics, and proteomics, are essential to elucidate the complex biological networks involved in MP-induced intestinal dysfunction and systemic metabolic disturbances.

In addition, the absence of standardized exposure assessment and analytical methodologies continues to limit accurate risk characterization and comparability across studies. Harmonized protocols for sampling, detection, physicochemical characterization, and quantification of MPs and NPs in environmental, food, and biological matrices are urgently required. Furthermore, the establishment of standardized exposure metrics—incorporating particle size, concentration, polymer composition, surface charge, and aging status—will be crucial for improving toxicological evaluation, regulatory decision-making, and human health risk assessment frameworks.

## 11 Conclusion

MPs have become ubiquitous contaminants within modern food systems, resulting in continuous human exposure through dietary intake. Experimental evidence from *in vitro* and animal studies suggests that MPs and NPs can adversely affect biological processes relevant to GI health by disrupting intestinal barrier integrity, inducing oxidative stress and inflammation, altering gut microbiota composition, and interfering with metabolic and immune homeostasis. Experimental studies also indicate that their ability to adsorb and transport toxic environmental contaminants may further amplify their biological effects.

However, despite substantial progress in experimental and mechanistic studies, direct evidence linking MP exposure to specific GI diseases in humans remains limited and inconclusive. Most available data are derived from *in vitro* systems and animal models, which may not fully replicate real-world human exposure scenarios. Therefore, although current findings support the biological plausibility of adverse GI and systemic effects, causal relationships between chronic dietary MP exposure and human disease have not yet been established. Consequently, the long-term health implications of chronic dietary exposure to MPs and NPs remain insufficiently understood, and current epidemiological evidence is not yet sufficient to quantify human health risks with confidence.

Future research should prioritize well-designed large-scale human epidemiological studies, standardized exposure assessment methodologies, and mechanistic investigations integrating microbiome, metabolomic, and immunological analyses. Such efforts are essential for establishing reliable risk assessment frameworks, clarifying the clinical relevance of MP exposure, and guiding evidence-based regulatory and mitigation strategies as the evidence base continues to evolve.

## Data availability statement

No new data were generated or analyzed in this review. Further inquiries can be directed to the corresponding author.

## Author contributions

ZB: Conceptualization, investigation, methodology, software, formal analysis, data curation, visualization, writing – original draft, writing – review & editing. RW: Conceptualization, data curation, formal analysis, investigation, methodology, project administration, resources, supervision, visualization, and writing – review & editing PE,.

## Generative AI statement

No generative AI tools were used for drafting or editing the manuscript text. Microsoft Copilot was used to assist in preparing the figures.

## Funding

Not applicable.

## Declaration of competing interest

The authors have nothing to declare.

## Abbreviations

AP-1, activator protein-1; γBB, γ-butyrobetaine; BPA, bisphenol A; CAT, catalase; C3G, cyanidin-3-glucoside; Cnt, canagliflozin; DEHP, di(2-ethylhexyl) phthalate; EFSA, European Food Safety Authority; FAO, Food and Agriculture Organization; FTIR, Fourier-transform infrared spectroscopy; GI, gastrointestinal; GMP, good manufacturing practices; GSH, glutathione; H_2_O_2_, hydrogen peroxide; HACCP, hazard analysis and critical control point; IBD, inflammatory bowel disease; IFN-γ, interferon-gamma; IL-6, interleukin-6; IL-1β, interleukin-1β; IRF-5, interferon regulatory factor-5; iSGLT2, inhibition of sodium-glucose cotransporter 2; LDH, lactate dehydrogenase; LDIR, laser direct infrared spectroscopy; LDPE, low-density polyethylene; LPS, lipopolysaccharide; MAPK, mitogen-activated protein kinase; MDA, malondialdehyde; MNPs, micro/nanoplastics; MPs, microplastics; MyD88, myeloid differentiation primary response 88; NET, neutrophil extracellular trap; NPs, nanoplastics; PBAT, poly(butylene adipate-co-terephthalate); PE, polyethylene; PET, polyethylene terephthalate; PFOS, perfluorooctane sulfonate; PLA, polylactic acid; PLGA, poly(lactic-co-glycolic acid); PMMA, polymethyl methacrylate; PP, polypropylene; POM, polyoxymethylene; PS, polystyrene; PVC, polyvinyl chloride; Py-GC/MS, pyrolysis-gas chromatography/mass spectrometry; ROS, reactive oxygen species; SGLT2, sodium-glucose cotransporter 2; SIRT1, sirtuin 1; SOD, superoxide dismutase; TCA, tricarboxylic acid; TLRs, Toll-like receptors; TNF-α, tumor necrosis factor-alpha; WHO, World Health Organization; ZO-1, zonula occludens-1.
